# Transient peripheral neuropathy following autologous breast reconstruction: a case series and evidence-based protocol for risk reduction

**DOI:** 10.1093/jscr/rjag376

**Published:** 2026-05-24

**Authors:** Denis Mulabdić, Christian Asher, Krzysztof Sosnowski, Charles M Malata

**Affiliations:** Department of Emergency Medicine, Dr. Josip Benčević General Hospital, Slavonski Brod, Ul. Andrije Štampara 42, 35000 Slavonski Brod, Croatia; Department of Plastic and Reconstructive Surgery, Norfolk and Norwich University Hospitals NHS Foundation Trust, Colney Lane, Norwich, NR4 7UY, United Kingdom; School of Clinical Medicine, University of Cambridge, Hills Rd, Cambridge CB20SP, United Kingdom; Department of Plastic and Reconstructive Surgery, Addenbrooke's Hospital, Cambridge University Hospitals NHS Foundation Trust, Hills Rd, Cambridge CB2 0QQ, United Kingdom; Anglia Ruskin University School of Medicine, Anglia Ruskin University, East Rd, Cambridge CB1 1PT, United Kingdom; Cambridge Breast Unit at Addenbrooke's Hospital, Cambridge University Hospitals NHS Foundation Trust, Hills Rd, Cambridge CB2 0QQ, United Kingdom

**Keywords:** peripheral neuropathy, neuropraxia, breast reconstruction, deep inferior epigastric perforator flap, intraoperative complications

## Abstract

Free flap reconstruction is the gold standard following mastectomy, yet peri-operative peripheral neuropathies remain underrecognized complications. This study aimed to identify peri-operative risk factors for upper limb peripheral nerve injury in patients undergoing autologous breast reconstruction and propose modifications to minimize risk. Out of 155 patients undergoing autologous abdominal breast reconstruction performed by a single surgeon between 2014 and 2022, eight developed transient postoperative upper limb neuropraxia. All affected patients were overweight/obese and classified as American Society of Anesthesiologists class II. Neuropraxia was most frequently ulnar or ulnar-median; 75% of symptoms resolved within two days. Half of the cases occurred ipsilateral to arterial line placement, with increased risk following multiple cannulation attempts. Prolonged operative duration and complex reconstructions contributed to neuropraxia risk. A five-step protocol was developed to mitigate nerve injury: ultrasound-guided arterial line placement; arm abduction <90°; forearm supination; meticulous padding of pressure points; early postoperative limb elevation and mobilization.

## Introduction

Autologous breast reconstruction following mastectomy, with a deep inferior epigastric perforator (DIEP) flap is regarded as the gold standard following mastectomy owing to its durable and natural aesthetic results [1, 2]. Despite pre-operative vascular imaging of the abdominal wall to expedite surgery, operative duration varies considerably depending on reconstructive requirements (e.g. unilateral versus bilateral versus bipedicled), previous abdominal surgery, irradiation within the anastomotic field, previous chemotherapy, and intra-operative challenges, result in variable operative time. Longer operative times are associated with increased morbidity; a recent study on bilateral DIEP flaps reported that each additional hour operating time increased morbidity by 19% [3]. Although uncommon, upper limb neuropathies are recognized complications of long operative times but have hitherto not been studied in the context of microvascular breast reconstruction [4, 5].

Peripheral nerves are well vascularized structures dependent on an anastomotic arterial network called the *vasa nervorum*. Elongation exceeding 5% of resting length can compromise arterial flow, resulting in transient ischaemia and neuropraxia of the nerve due to kinking and narrowing of arterioles [6]. Traction and external nerve compression are the predominant causes of post-operative peripheral nerve injury [7]. Stretch-related upper limb neuropraxia typically follows sustained positioning at the end range of a joint, including ulnar nerve elongation with elbow flexion ˃100° [7]. Furthermore, arm abduction below 30° or ˃90° poses a greater risk for ulnar nerve compression [8]. Compression can be caused by routinely-used external devices including poorly fitting blood pressure cuffs or sustained arm positioning on a board with inadequate padding [9].

The monitoring and control of haemodynamic parameters during microvascular breast reconstructive surgery often necessitates arterial line placement in preference to external blood pressure cuffs. Although radial artery cannulation carries a relatively low complication rate, its proximity to the median, radial, and ulnar nerves, however, makes it a potential cause of iatrogenic injury, either directly or via secondary oedema or haematoma. The risk increases with multiple unsuccessful canulation attempts [10].

The typical protocol for intraoperative patient positioning during autologous breast reconstruction with lower abdominal free flaps [be they DIEP or superficial inferior epigastric artery (SIEA)] places the patient in a relaxed ‘crucifix’ position: supine and central on the table, with both arms extended on padded arm boards, shoulders abducted short of 90°, the patient’s waist sited over the break point of the operating table and the knees gently flexed over a pillow. All bony pressure points are liberally padded with gel or cotton gamgee as consistent pressure of only 70 mm Hg applied to bony prominences for 2 h or longer may cause irreversible tissue ischemia [11]. Further considerations should be taken for patients who are at greater risk for pressure injuries. In this context, viscoelastic polymer pads for limb support are regarded as a superior alternative to standard operating table mattresses, due to enhanced pressure redistribution properties and higher skin contact [12, 13]. Nimalan *et al.* encourage passive movement of joints throughout and at the end of surgery to promote circulation to optimize functional outcomes and reduce the risk of injury [14]. As such prioritizing movement of joints that are positioned outside of relaxed anatomical position (shoulders, elbows, and wrists) every 2 h is prudent. Postoperatively, early mobilization within the first 24 h and physiotherapy as early as possible improves muscle strength and mobility, whilst decreasing duration of hospital stay in breast reconstruction patients [15].

Despite adherence to positioning and padding protocols, patients continue to report transient upper limb symptoms consistent with neuropraxia following microvascular breast reconstruction. To address this, we reviewed our institutional experience to identify peri-operative risk factors contributing to nerve injury and, based on these findings, developed a five-step evidence-based protocol designed to minimize upper limb neuropraxia and enhance patient safety.

## Case series

All patients who reported neurological symptoms following autologous breast reconstruction with abdominal free (DIEP/ SIEA) flaps by a single surgeon in a tertiary university centre between January 2014 and June 2022 were evaluated with respect to their demographics, duration of symptoms, operative details, and postoperative outcomes.

Of the 155 DIEP/SIEA free flap reconstructions performed, eight patients reported postoperative symptoms in keeping with neurological disturbances of upper limb peripheral nerves. Patient demographics including age, American Association of Anaesthesiologists Classificaton (ASA) classification, and body mass index (BMI) are detailed in Table 1.

**Table 1:** Patient demographics.

Patient	Date	Age	ASA	BMI
1	12/2015	42	II	25
2	02/2016	32	II	26
3	03/2016	57	II	32
4	07/2016	40	II	29
5	08/2017	43	III	36
6	12/2017	34	II	25
7	02/2022	48	II	29
8	05/2022	57	II	33

The eight patients in the series included one case of salvage bilateral DIEP reconstruction, whilst the remaining seven were immediate reconstructions consisting of four bilateral, one unilateral reconstruction using two stacked DIEP’s, and two bi-pedicled DIEP reconstructions (see Table 2). The mean operative time was 14.1 ± 1.3 h. The standard relaxed crucifix position was adopted with gel pads and gamgee to alleviate pressure. Arterial lines were placed for monitoring in all cases, six sited within the radial artery, one within the dorsalis pedis and one within the brachial artery.

**Table 2:** Summary of operative characteristics.

Patient	Mastectomy laterality	Immediate, delayed or salvage	Reconstruction	Length of surgery (hours)
1	Bilateral mastectomy	Immediate	Hemi-DIEP flaps.	11.5
2	Right mastectomy and axillary clearance	Immediate	Bi-pedicled DIEP flap	13
3	Bilateral mastectomy	Salvage	Ipsilateral DIEP flaps	14
4	Right mastectomy and axillary clearance	Immediate	Side-by-side bilateral hemi-DIEP flaps	14
5	Bilateral Wise-pattern mastectomies	Immediate	Bilateral DIEP flaps	15
6	Left mastectomy	Immediate	Bi-pedicled DIEP flap	14
7	Bilateral	Immediate	Bilateral DIEP flaps	16
8	Bilateral mastectomies	Immediate	Hemi-DIEP flaps	15.5

Four radial artery lines were placed on the right and two on the left. Three patients experienced ulnar nerve paraesthesia ipsilateral to where the radial arterial line had been attempted, all requiring three attempts at placement. One patient experienced symptoms ipsilateral to a single unsuccessful attempt at a radial arterial line. Patients reported symptoms within one post-operative day, with 75% (6/8) resolving by the second postoperative day. A total of 62.5% (5/8) reported ulnar nerve symptoms, 37.5% (3/8) had combined ulnar and median nerve symptoms. The left hand was affected in 37.5% (3/8), the right hand in 50.0% (4/8), and bilaterally in 12.5% (1/8) (see Table 3). All eight patients were overweight (BMI >25.0 kg/m^2^) with an average BMI of 29.4 ± 3.8, three patients were obese (BMI >30 kg/m^2^) (Table 1). Seven patients (88%) were within the ASA classification II, and one patient (12%) III (see Table 1). All cases were anaesthetized by consultant anaesthetists with a specialist interest in patients undergoing in free flap breast reconstruction.

**Table 3:** Line characteristics and neuropraxia outcomes.

Patient	Location of lines	Laterality of lines	Attempts at line insertion	Patient positioning	Neuropraxia laterality	Nerve distribution	Duration of neuropraxia
1	Radial	Right	1	Standard	Right	Ulnar	3 days
2	Brachial	Left	3	Standard	Left	Combined (Median + Ulnar)	2 days
3	Brachial	Right	2^a^	Standard	Left	Ulnar	2 days
4	Radial	Right	1	Standard	Right	Combined (Median + Ulnar)	2 days
5	Radial	Left	1	Standard	Right	Ulnar	2 days
6	Radial	Right	3	Standard	Right	Combined (Median + Ulnar)	12 h
7	Dorsalis Pedis	Left	3^b^	Standard	Bilateral	Ulnar	2 days
8	Radial	Left	3	Standard	Left	Ulnar	2 days

^a^1^st^ attempt radial.

^b^Attempts 1^st^ right radial, 2^nd^ left radial.

## Discussion

Symptoms of neuropathy include paraesthesia and weakness in the distribution of the affected nerves. The correlation between upper limb neuropraxia and anaesthesia was initially outlined by Büdinger in 1894 [16]. Today, known risk factors include co-morbidities such as type 2 diabetes mellitus, hypertension, and hypothyroidism, previous peripheral neuropathy, extremes of BMI and intra-operatively, the location of pressure cuffs, arm position, and duration of surgery [17]. In the present case series, 8 of the 155 patients reported symptoms confined to the hand and their short duration suggests a reversible aetiology (neuropraxia).

The present study also illustrates three risks for post-operative neuropraxia in this cohort of patients. A raised BMI facilitating wholly autologous breast reconstruction from surplus abdominal tissue was a consistent feature amongst patients with neuropraxia. Longer operative times associated with more complex reconstructive cases, including bilateral free flap breast reconstruction and salvage reconstruction, presenting numerous challenges [18]. Iatrogenic precipitants of swelling and inflammation in a relatively inelastic, immobilized limb. Most cases in this cohort of patients had symptoms of neuropraxia ipsilateral to the location of arterial or attempted arterial line placement.

The lack of permanent nerve symptoms highlights our current protocol, in particular; avoiding excessive arm abduction and meticulous padding of the elbows is effective in reducing the risks of surgery despite the prolonged duration of surgery. The length of reconstructive microvascular breast reconstructive surgery is a crucial determinant of morbidity as recently reported by Haddock *et al.*, stratifying incidence of complications according to procedural time [3]. Procedures lasting <5 h had 9.5 times fewer postoperative complications than those lasting ≥5 h. A 7–9-h procedure had 5.5 times fewer complications over procedures lasting ≥9 h [3].

Mitigation of risks starts with risk identification pre-operatively and implementation of modifications to enhance the standard protocols with the aim of reducing incidence and severity.

Patients at highest risk (higher BMI and predicted longer operative times; bilateral breast reconstruction and salvage reconstruction and bipedicled flap reconstruction) can be counselled pre-operatively regarding risk of peripheral nerve damage as part of the consent process. They can be reassured that these symptoms are likely to be transient and informed of the interventions that will minimize the risk of incidence and duration.

The placement of arterial lines, where indicated, can be optimized with the use of ultrasound [19]. Cho *et al.* demonstrated it reduced procedural time as well as postoperative complications, particularly in patients with elevated BMI [20].

With natural transition points during surgery, limb positioning can be modified without adding delay to operative time. These include the immediate period following mastectomy where there may be a complete change of instrumentation and scrub practitioner, followed by haemostasis, mastectomy flap, and pocket assessment by the reconstructive surgeon. Similar potential breaks in the operation are the interval between internal mammary recipient vessel preparation and commencement of microsurgical anastomosis and that prior to abdominal closure and flap inset following completion of microsurgical anastomosis. The upper limb position can be further optimized by maintaining supination if anatomically possible at all stages of surgery. Hewson *et al.* note that arm pronation results in greater humeral rotation increasing contact and pressure between the structures in the ulnar groove, when compared to supination [21]. As such neutral or supination is recommended when the patient is supine, with neutral positions preferred when the patient’s arms are fully adducted alongside the torso.

In addition to arm positioning, the choice of operative surface significantly influences peripheral nerve safety. Conventional two-inch elastic foam mattresses, though stable, may increase localized pressure and nerve compression. Multilayer viscoelastic supports provide superior pressure distribution, increasing contact area by ~60% [22]. For high-risk patients, their use—combined with meticulous padding and periodic intraoperative limb adjustments—may further reduce compression-related neuropraxia.

## Conclusions

The main strength of this study is the integration of detailed intraoperative positioning strategies with postoperative monitoring guidelines into a practical, evidence-based protocol designed to reduce neuropathic complications in autologous breast reconstruction. The inclusion of multiple consecutive cases allowed for the identification of consistent procedural risk factors and clinical outcomes. However, the study is limited by its relatively small sample size and retrospective design, which may limit the generalizability of the findings. Additionally, electrophysiological follow-up was not available for patients. Despite these limitations, the observed outcomes strongly support the clinical relevance of the proposed protocol.

We itemize five interventions to further augment current peri-operative practice, based on the evidence underpinning the common themes identified in this cohort of patients.

(1) Routine use of ultrasound in radial artery line placement should be considered, particularly in higher risk patients.
(2) Abduction of arms below 90°, with relaxation of high abducted arms at regular 2-h intervals intra-operatively.
(3) Forearms preferentially positioned in supination.
(4) Meticulous padding of all pressure points with consideration for type of padding used.
(5) Immediate post-operative upper limb oedema management: forearm elevation on pillows and day 1 post-operative mobilization and physiotherapy.

Implementation and adherence to this protocol offers a wholistic multi-disciplinary approach to improving patient outcomes. Its implementation may minimize both the incidence and duration of peri-operative peripheral nerve injury in patients undergoing complex microvascular breast reconstruction.
